# Intrabronchial Valve Treatment for Prolonged Air Leak: Can We Justify the Cost?

**DOI:** 10.1155/2016/2867547

**Published:** 2016-04-04

**Authors:** Eitan Podgaetz, Felix Zamora, Heidi Gibson, Rafael S. Andrade, Eric Hall, H. Erhan Dincer

**Affiliations:** ^1^Division of Cardiothoracic Surgery, Section of Thoracic and Foregut Surgery, University of Minnesota, MMC 207, 420 Delaware Street, SE, Minneapolis, MN 55455, USA; ^2^Division of Pulmonary, Allergy, Critical Care and Sleep Medicine, University of Minnesota, MMC No. 276, 420 Delaware Street, SE, Minneapolis, MN 55455, USA; ^3^Cardiopulmonary Services, Endoscopy Department, University of Minnesota, MMC No. 247, 500 Harvard Street, SE, Minneapolis, MN 55455, USA; ^4^University of Minnesota, Financial Office, 500 Harvard Street, SE, Minneapolis, MN 55455, USA

## Abstract

*Background.* Prolonged air leak is defined as an ongoing air leak for more than 5 days. Intrabronchial valve (IBV) treatment is approved for the treatment of air leaks.* Objective.* To analyze our experience with IBV and valuate its cost-effectiveness.* Methods.* Retrospective analysis of IBV from June 2013 to October 2014. We analyzed direct costs based on hospital and operating room charges. We used average costs in US dollars for the analysis not individual patient data.* Results.* We treated 13 patients (9 M/4 F), median age of 60 years (38 to 90). Median time from diagnosis to IBV placement was 9.8 days, time from IBV placement to chest tube removal was 3 days, and time from IBV placement to hospital discharge was 4 days. Average room and board costs were $14,605 including all levels of care. IBV cost is $2750 per valve. The average number of valves used was 4. Total cost of procedure, valves, and hospital stay until discharge was $13,900.* Conclusion.* In our limited experience, the use of IBV to treat prolonged air leaks is safe and appears cost-effective. In pure financial terms, the cost seems justified for any air leak predicted to last greater than 8 days.

## 1. Introduction

Both minimally invasive surgical approaches and bronchoscopic therapies to treat prolonged air leaks have regained interest from the medical and surgical communities.

Air leaks, both alveolopleural (APF) (lung parenchyma to pleura) and bronchopleural (BPF) (bronchus to pleura) fistulas may occur after surgical lung resection, such as a wedge biopsy, segmentectomy, or lobectomy as well as iatrogenic or spontaneously in those with underlying pulmonary disease. The incidence of postoperative air leak ranges from 28% to 60% immediately after surgery, 26% to 48% on postoperative day 1, 22% to 24% on day 2, and 8% on day 4 [1–3].

Primary spontaneous pneumothoraces (PSP) are usually seen in people aged 20 to 30 with an incidence of 7.4–18 cases per 100,000 per year for men and 1.2–6 cases per 100,000 per year for women while secondary spontaneous pneumothoraces (SSP) occur in patients aged 60–65 with incidence of 6.3 cases per 100,000 per year for men and 2.0 cases per 100,000 per year for women [4, 5].

Incidence of iatrogenic pneumothorax is reported as 1.36% in hospitalized patients due to invasive procedures or positive pressure ventilation [6].

Most experts consider air leak to be prolonged if they persist beyond 5 days of diagnosis. Treatment options for prolonged air leak include noninvasive and invasive techniques. Noninvasive approaches rely on prolonged chest tube drainage either on water seal or Heimlich valve system or coupled with ventilator strategies to establish acceptable ventilation while reducing the flow through the alveolo- or bronchopleural fistula [7].

Invasive therapy options include pleurodesis either surgical or at bedside through the indwelling chest tube, by instillation of talc slurry or doxycycline, mechanical pleurodesis by pleural abrasion, application of fibrin sealant, bronchial stump stapling or resuturing, reinforcement with muscle, pleural, omental, or pericardial fat pad flap to the bronchial stump, and in some patients completion of surgical lobectomy. In recent years, bronchoscopic treatment for complex alveolopleural [7–9] and bronchopleural fistulas [10, 11] with IBV valves has been published as small case series or in review articles.

The IBV system (Spiration, Redmond, WA, USA) has been approved under the Humanitarian Device Exemption (HDE) program for prolonged air leaks after segmentectomy, lobectomy, and lung volume reduction surgery on October 24, 2008.

We describe our experience with IBV valve system in patients with prolonged air leak and perform a cost valuation to better understand and justify its true cost when used in selected patients in a cost conscious environment.

## 2. Patients and Methods

### 2.1. Patient Selection

From June 2013 through October 2014, 13 patients with prolonged alveolopleural air leak underwent IBV valve treatment at our institution. A member of our multidisciplinary Complex Airway Center saw all patients and decision for valve treatment was made jointly after considering invasive and noninvasive options. All patients who presented with prolonged air leaks during the time period regardless of the etiology were included in the study, not just patients in whom balloon occlusion technique was successful at the time of the procedure. All patients had tube thoracostomy at the time the air leak/pneumothorax was identified.

### 2.2. Bronchial Valve and Procedure

**Figure 1:**
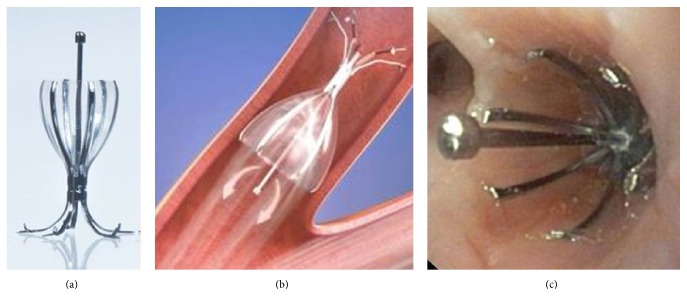
(a) Intrabronchial valve (IBV), (b) deployed IBV schematic, and (c) intraoperative photo of deployed IBV.

The Spiration IBV system is an umbrella-shaped, self-expanding device with a nickel-titanium (Nitinol) frame. The valve is secured to the airway with 5 distal anchors, while 6 proximal struts hold the membrane apposed to the airway wall (Figure 1). This unidirectional valve blocks air entry distal to the valve while allowing secretion and air to escape around the membrane proximally into the central airways. A central rod facilitates grasping with forceps for removal if necessary and sometimes to reposition.

Bronchoscopy was performed through an endobronchial tube under general anesthesia in all patients. We used all currently available sizes of valves, 5, 6, and 7 mm, uncompressed. The first step included a thorough visual airways assessment. Then using standard occlusion Fogarty catheters (Edwards Lifesciences, Irvine, CA) we systematically perform segmental isolation until the exact location of the air leak was identified. We start by isolating main bronchi and then lobar bronchus and finally segmental airways. Sustained Valsalva to a pressure of 40 cm H_2_O is performed during isolation maneuvers to prove that the air leak is not present with balloon isolation. Once localized, a calibrated balloon catheter is used to size the airways using standardized visual assessment (B5-2C, Olympus America, Center Valley, PA). The valves are then deployed via a catheter in which the valve is compressed inside and can pass through a flexible bronchoscope with a ≥2.6 mm channel (Olympus T-190).

### 2.3. Cost Analysis

We compared cost associated with the median hospital stay in days for our cohort based on the level of care during their stay (room and board at floor, intermediate care, or ICU level) and multiplied by the cost for each different level of service. We then compared that amount to the costs associated with implantation of the IBV. Each patient's information before and after IBV placement was used for direct cost comparison.

During the analysis, costs other than room and board, operating room, and anesthesia charges were excluded, including costs related to patient care such as imaging studies (CXR and CT scans), laboratory test ordered, medication charges, and ward visits including physical and occupational therapy. It is difficult to standardize cost analysis in such a heterogeneous population, hence the rationale to exclude other confounder data.

### 2.4. Follow-Up

All patients received a chest X-ray (CXR) after the procedure and then daily CXR for management of their chest tubes until discharge.

## 3. Results

### 3.1. Patient Demographics and Characteristics

There were 13 patients treated with IBV for prolonged air leaks. Patient characteristics and demographics are shown in Table 1.

All 13 patients had alveolopleural fistulae and were treated with 100% success rate and removal of chest tubes regardless of the underlying cause for air leak.

Among this cohort, 4 patients had iatrogenic pneumothorax with prolonged air leak secondary to CT guided lung biopsy (cases 3, 7, and 10) and pacemaker placement (case 12) and 7 patients had secondary spontaneous pneumothorax either in presence of COPD (2, 5, 9, and 13) or in presence of metastatic lung cancer (cases 1, 6, and 8). The remaining 2 patients had postoperative air leaks resulting from a pulmonary wedge resection (case 4) and from a thoracoscopic lobectomy (case 11).

Three unique patients had secondary spontaneous pneumothorax as a result of pulmonary metastatic disease from angiosarcoma, pancreatic cancer, or osteosarcoma. The time to resolution of the air leak and eventually chest tube removal was significantly higher in them than in the rest of the cohort. The days (median) for the air leak resolution and chest tube discontinuation were 9 and 11 days versus 2 and 3 days, respectively.

Intrabronchial valves were not removed in patients with metastatic lung cancer where expected survival was less than 6 months. All other patients were seen 4–6 weeks later for bronchoscopic valve removal.

There was no valve related complication during placement, follow-up, or removal procedures. All the valves were intact at the time of removal without any damage, distortion, or missing parts.

The median hospital stay for floor care was 8 days, intermediate care 9.4 days, and ICU stay 1.4 days.

The direct costs associated with the room and board for the level of care provided at our institution are $575.34 for floor care, $891.531 for intermediate care, and $1142.33 for ICU care.

The total cost of the hospitalization (room and board) for the average patient in our cohort was $14,605.22, of which on average $12,303 (85%) were spent before IBV implantation.

The IBV cost is $2750 per valve. The average number of valves used per patient was 4 for a median cost of $11,000 plus OR and anesthesia time of $599 for the first 30 minutes with an increase $558 for each additional 15 minutes of procedure time, for a total of $11,599. Average procedure duration was under 30 minutes. Adding the costs of the IBV and procedure time to the 4 days until discharge, the average total cost was $13,900 from the time of IBV placement to discharge.

## 4. Discussion

Patients with prolonged air leaks, regardless of the cause, remain to be a challenge to treat. Our 16-month experience with Spiration® IBV system treating 13 patients with prolonged air leaks from various etiologies (status after lung resection, secondary spontaneous pneumothorax, and iatrogenic pneumothorax) was reviewed.

Endobronchial and intrabronchial valves (EBV and IBV) rely on the fact that a unidirectional valve will prevent air entry during inspiration while allowing expiratory airflow and drainage of secretions [1, 2, 4–6]. EBV are only approved for use in Europe at the time of this publication under the name Zephyr® made by Pulmonx.

Closure of a BPF by using an endobronchial valve has been reported in the past for alveolopleural fistulas [7–9].

To date, only case series have been published in the treatment of prolonged air leak using IBV or EBV. First, Firlinger et al. reported a series of 16 patients with prolonged air leak whom chest tubes remained in place for at least 7 days who underwent valve placement after balloon occlusion. The source of air leak was endoscopically identified in 13 patients (81%) and 3 of them did not respond to the valve placement due to persistent air leak requiring other interventions later on. All nonresponders and 7 of 10 responders were treated with IBV valve while others received EBV valve [12].

Other series by Gillespie et al. showed improvement in air leak in 7 patients by using IBV with a median duration of air leak of 4 weeks before and 1 day after treatment and a mean of 4.5 days [8].

Cordovilla et al. implanted valves by flexible bronchoscopy in 8 patients; a median of 2 valves (1–4) was used, with a median duration of air leak prior to placement of 15.5 days. The achieved complete resolution of air leak in 75% of patients is with a median duration of drainage after insertion of the valves of 13 days and a median time to valve removal of 52.5 days [13].

A series by Dooms et al. looked at the use of IBV for the treatment of air leak following anatomical pulmonary resection (APF). They included 10 patients out of 277 anatomical resections over 16-month period. They saw resolution of the air leak in a median of 2 days and chest tube removal at 4 days [9].

It is evident that our cohort is composed of a very heterogeneous group and this makes it difficult to draw definitive conclusions. It does represent one of the largest experiences with IBV reported to date and is the first to look at the costs of IBV, which will be valuable information for practicing clinicians and administrators alike [14].

The patients with secondary spontaneous pneumothorax due to metastatic cancer experienced longer duration for the air leak after valve placement, with mean of 9 days as compared to only 2 days in other patients with iatrogenic pneumothorax and secondary spontaneous pneumothorax in the presence of COPD or after lung resection. While we do not have a proven reason for this, these patients had multiple diffuse lung metastatic lesions with partial response to chemotherapy causing tumor necrosis and alveolopleural fistulae. IBV was selected as the patients had failed all other therapies (chemical and/or surgical pleurodesis or blood patch prior to valve placement) and it was decided under our multidisciplinary airway program to use them under compassionate use care. We attempted to treat the most prominent lesion seen on CT scan but there could have been other lesions contributing to the air leak despite performing balloon occlusion maneuvers.

We performed balloon test occlusion to identify the location of the air leak in patients with secondary spontaneous pneumothorax, but in patients with a known location based on CT scan findings or postsurgical, the valves were deployed without the need for balloon test occlusion; this explains why our average procedure times are under 30 minutes and deployment of IBV into a known location takes less than 10 minutes. The average number of valves used was 4 per patient (2 to 6 valves for any given patient) depending upon the lobe(s) treated. An important point to mention is that many of the published series are with endobronchial valves (EBV, Zephyr made by Pulmonx) which are currently only approved for use in Europe and not USA. These valves can accommodate up to 8.5 mm airway. In the US, the FDA approved intrabronchial valves (IBV, Spiration made by Olympus) which only come in sizes 5 mm, 6 mm, and 7 mm; hence in many instances we could have used a larger valve to occlude a more proximal segment but in order to be completely occlusive we decided to size down and place the valves in subsegments. This means that a direct comparison between number of valves deployed between EBV and IBV is not objective.

We did not encounter any procedural or valve related complications. We decided to leave valves in 3 cancer patients and 1 COPD patient whom expected survival was predicted as 6 months or less due to their diseases.

To our knowledge none of our patients has been readmitted with a recurrent pneumothorax or pleural space problem.

Despite our success treating prolonged air leaks with IBV, we acknowledge several limitations in our study. First, this represents a small group of diverse patients; we do not have a true control group and our cost analysis is somewhat crude in the sense that many of the peripheral costs were excluded and we used medians from a very heterogeneous group to compare costs.

All patients except one had a prolonged air leak (6 to 88 days) prior to treatment with IBV. We decided to place the valves early at day 2 of air leak in one patient with metastatic angiosarcoma to the lung based on our previous experience with other metastatic cancer patients and again as a last resort under compassionate care use. Some of our patients were transferred from outside institutions after many days of unresolved air leak and, while in our institution, we were not notified of some patients' case until after many days as well.

It is difficult to recommend establishing treatment guidelines for the use of IBV based on our experience. Based exclusively on cost, IBV implantation and postoperative care costs alone are comparable to the cost for a hospitalized patient after 8 days with an unresolved air leak. Clearly we do not have a prognostic score to predict which patient will have an air leak that will last until day 8, and in fact many air leaks resolve spontaneously before that. Clinical experience, clear judgment of the etiology of the air leak, the characteristics of the underlying lung parenchyma, and the nutritional status of the patient is essential to make that determination. Currently we use traditional analog chest drainage systems connected to the chest tube measuring bubbles in a chamber to determine the air leak, but perhaps a digital pleural drainage system may be able to help understand air leaks better and help decide on which patients IBV implantation would make more sense, as described by Dooms et al. [9]. We did not quantify the severity of the air leak based on the analog system.

We cannot ignore that other alternatives to treat air leaks exist such as pleural tents, mechanical or chemical pleurodesis, or discharging patient's home with a one-way Heimlich valve and then removing the tube when the air leak has subsided in clinic usually 1–3 weeks later. The costs related to all other strategies were not analyzed or compared to perform a more robust cost efficacy study. Based on our data it appears that the cost is less when IBVs are used at a timely manner in properly selected patients. We also did not dwell on potential complications from these other techniques, some which have been reported fatal. This study also fails to include all the nondirect associated and potential costs related to having an indwelling chest tube for a longer time, including clinic visits, imaging studies on occasion antimicrobial treatment and the much ignored negative effect on quality of life, earlier return to activity (work/school), productivity days lost, potential for infection (empyema), and overall patient satisfaction which are frequently hard to measure and quantify.

Considering our success rate being 100% both in deployment accuracy and cessation of the air leak, along with a very safe profile one can speculate that the intrabronchial valve treatment might be considered in patients with prolonged air leaks, possibly allowing them to be discharged home sooner without a chest tube, minimizing potential infection risks with prolonged chest tube drainage, and increasing patient satisfaction at least by personal reports from our cohort when compared to patients we used to send home on Heimlich valves. In carefully selected patients there will also be a financial benefit as it can minimize direct hospital costs and eliminate the risk of other potential complications to occur while hospitalized while providing faster return to activities and work, reducing productivity days lost.

Further studies are needed to find out the ideal time of valve placement and the ideal patient in whom to use it in order to avoid prolonged air leaks and minimize expenses. Ideally coming up with an air leak prognostic score may be of utility to decide in whom and when to implant the valves. These studies should be able to help us not only understand the impact of IBV on patient satisfaction and quality of life but also clarify if this strategy is truly cost-effective and worthwhile.

## 5. Conclusion

The intrabronchial valve system is a very safe and effective method of treating patients with prolonged air leak from virtually any etiology. We encountered early discharge from the hospital after implantation, allowing quick recovery of the patients while minimizing potential infection risks well known for patients with chronic indwelling chest tubes. We found that the cost of IBV can be justified in selected patients, especially if used earlier when one can avoid the costs of a longer hospital stay. Direct cost comparisons with other treatments are needed to better understand the cost-effectiveness in comparison to other alternatives. Further studies are underway to understand the role of IBV therapy and how to properly select the correct patient population who may benefit from its use.

## Figures and Tables

**Table 1:** Patient characteristics and demographics.

Patients	Age/gender	Reason for procedure	Type	Underlying lung disease	Lobe(s) treated	Leak duration before IBV	Valves placed (*n*)	Air leak resolved/chest tube removal (days)
1	48/M	Air leak	APF	SSP, osteosarcoma	LUL, Lingula	88	6	9, 11
2	60/M	Air leak	APF	SSP, COPD	RUL	6	4	7, 9
3	51/F	Air leak	APF	COPD, iatrogenic	RUL	11	3	1, 2
4	79/M	Air leak	APF	After wedge	LUL	9	4	3, 5
5	63/M	Air leak	APF	SSP, COPD	RUL	15	3	1, 2
6	75/M	Air leak	APF	SSP, angiosarcoma	RLL	2	5	35, 45
7	81/M	Air leak	APF	COPD, iatrogenic	LUL	8	5	1, 2
8	38/F	Air leak	APF	SSP, pancreatic CA	RLL	8	4	2, 3
9	48/M	Air leak	APF	SSP, COPD	LUL	10	6	6, 7
10	51/F	Air leak	APF	COP, iatrogenic	RML	9	2	1, 2
11	56/M	Air leak	APF	After lobectomy	Lingula	10	2	2, 3
12	90/F	Air leak	APF	COPD, iatrogenic	LUL	7	4	2, 3
13	65/M	Air leak	APF	SSP, COPD	RUL	11	4	2, 3

SSP: secondary spontaneous pneumothorax.

COP: cryptogenic organizing pneumonia.

APF: alveolopleural fistula.

BPF: bronchopleural fistula.

## References

[1] Okereke I., Murthy S. C., Alster J. M., Blackstone E. H., Rice T. W. (2005). Characterization and importance of air leak after lobectomy. *Annals of Thoracic Surgery*.

[2] Okamoto J., Okamoto T., Fukuyama Y., Ushijima C., Yamaguchi M., Ichinose Y. (2006). The use of a water seal to manage air leaks after a pulmonary lobectomy: a retrospective study. *Annals of Thoracic and Cardiovascular Surgery*.

[3] Alphonso N., Tan C., Utley M. (2005). A prospective randomized controlled trial of suction versus non-suction to the under-water seal drains following lung resection. *European Journal of Cardio-thoracic Surgery*.

[4] Melton L. J., Hepper N. G. G., Offord K. P. (1979). Incidence of spontaneous pneumothorax in Olmsted county, Minnesota: 1950 to 1974. *American Review of Respiratory Disease*.

[5] Gupta D., Hansell A., Nichols T., Duong T., Ayres J. G., Strachan D. (2000). Epidemiology of pneumothorax in England. *Thorax*.

[6] Celik B., Sahin E., Nadir A., Kaptanoglu M. (2009). Iatrogenic Pneumothorax: Etiology, Incidence and Risk Factors. *The Thoracic and Cardiovascular Surgeon*.

[7] Kempainen R. R., Pierson D. J. (2001). Persistent air leaks in patients receiving mechanical ventilation. *Seminars in Respiratory and Critical Care Medicine*.

[8] Gillespie C. T., Sterman D. H., Cerfolio R. J. (2011). Endobronchial valve treatment for prolonged air leaks of the lung: a case series. *Annals of Thoracic Surgery*.

[9] Dooms C. A., Decaluwe H., Yserbyt J., De Leyn P., Van Raemdonck D., Ninane V. (2014). Bronchial valve treatment for pulmonary air leak after anatomical lung resection for cancer. *European Respiratory Journal*.

[10] Mahajan A. K., Doeing D. C., Hogarth D. K. (2013). Isolation of persistent air leaks and placement of intrabronchial valves. *Journal of Thoracic and Cardiovascular Surgery*.

[11] Mahajan A. K., Verhoef P., Patel S. B., Carr G., Hogarth D. K. (2012). Intrabronchial valves: a case series describing a minimally invasive approach to bronchopleural fistulas in medical intensive care unit patients. *Journal of Bronchology and Interventional Pulmonology*.

[12] Firlinger I., Stubenberger E., Müller M. R., Burghuber O. C., Valipour A. (2013). Endoscopic one-way valve implantation in patients with prolonged air leak and the use of digital air leak monitoring. *Annals of Thoracic Surgery*.

[13] Cordovilla R., Torracchi A. M., Novoa N. (2015). Endobronchial valves in the treatment of persistent Air Leak, an alternative to surgery. *Archivos de Bronconeumologia*.

[14] Podgaetz E., Andrade R., Zamora F. D., Gibson H., Dincer H. (2015). Cost valuation of intrabronchial valve (IBV) treatment for air leak. *American Journal of Respiratory and Critical Care Medicine*.

